# Human Factors in the Cybersecurity of Autonomous Vehicles: Trends in Current Research

**DOI:** 10.3389/fpsyg.2019.00995

**Published:** 2019-05-03

**Authors:** Václav Linkov, Petr Zámečník, Darina Havlíčková, Chih-Wei Pai

**Affiliations:** ^1^Department of Traffic Psychology, CDV – Transport Research Centre, Brno, Czechia; ^2^Graduate Institute of Injury Prevention and Control, College of Public Health, Taipei Medical University, Taipei, Taiwan

**Keywords:** autonomous vehicle, cybersecurity, human factor, cyberattack, hackers

## Abstract

The cybersecurity of autonomous vehicles (AVs) is an important emerging area of research in traffic safety. Because human failure is the most common reason for a successful cyberattack, human-factor researchers and psychologists might improve AV cybersecurity by researching how to decrease the probability of a successful attack. We review some areas of research connected to the human factor in cybersecurity and find many potential issues. Psychologists might research the characteristics of people prone to cybersecurity failure, the types of scenarios they fail in and the factors that influence this failure or over-trust of AV. Human behavior during a cyberattack might be researched, as well as how to educate people about cybersecurity. Multitasking has an effect on the ability to defend against a cyberattack and research is needed to set the appropriate policy. Human-resource researchers might investigate the skills required for personnel working in AV cybersecurity and how to detect potential defectors early. The psychological profile of cyber attackers should be investigated to be able to set policies to decrease their motivation. Finally, the decrease of driver’s driving skills as a result of using AV and its connection to cybersecurity skills is also worth of research.

## Introduction

Autonomous vehicles (AV) are vulnerable to many kinds of cyberattacks. The software driving fully AV will have more than 100 million lines of code, so it is impossible to predict the security problems (Parkinson et al., 2017). It is important to study the different ways to attack an AV, the ways to reduce the probability of attacks, and how to minimize the damage. The human is always the weakest point in defending against an attack and dealing with the consequences; therefore, reducing human-induced errors is most effective. Preventing human failure should be taken into account when designing AV (Chong et al., 2018). This is an opportunity for human-factor researchers and psychologists to improve the cybersecurity practices of AV (Proctor and Chen, 2015). In this text, we review how better to protect AV from cyberattacks. First, we discuss the kinds of cyberattacks to which AV is susceptible with focus on those types of attacks where human factor is important. Second, we review the topics studied by psychologists and human-factor researchers to improve cybersecurity and what might be done specifically for AV.

## Cybersecurity Issues in Autonomous Vehicles

There are many ways to initiate an AV cyberattack. An attack can target the software that manages visual information and road infrastructure, or it could be a physical attack on the vehicle’s hardware (Lima et al., 2016). An attack on the remote keyless entry might lock a person inside the car or prevent locking at all (Checkoway et al., 2011). If tire-pressure monitor systems are under the control of an attacker, they might present false readings and hide regular air pressure leakage reduction. An attack on the inclination sensor might cause the car to slow down or start to brake because the sensor signals a steep gradient (Parkinson et al., 2017).

Car communication could be susceptible. The attack could be active, like when the communication is interrupted or replaced by false messages, or it could be passive, like when the attacker gathers information in long-term period for a future malicious purpose (like selling information to some company). Even when the listener cannot decode the data, the time of day the driver uses the car or where the car is located might be still abused (He et al., 2017). There are several types of active attacks on car communication. A spoofing attack is where the attacker uses a false identity or sends false data (e.g., they can pretend that they are a neighboring car or send false information about the neighboring car location). A man-in-the-middle attack is where the attacker gets the original message sent to the car, changes it, and sends the new message to the car (He et al., 2017). A denial of service attack is where the attacker sends a large amount of data to the car so that the communication channel is blocked (Bergin, 2015). Jamming is where a background radio noise blocks the frequency used for communication (Parkinson et al., 2017). A black hole attack is where a message is blocked without informing the car about the missing message (Bergin, 2015). Other types of attacks on AV communication include falsifying the sender’s digital signature, forcing the car to restart, and replacing the car communication certificate with a false one (Petit and Shladover, 2014).

The human factor is central for other types of AV attacks. The car information system might be infected by malware, which can cause future damage (Takahashi, 2018), and a human mistake is the most probable source of the infection (e.g., people might download it from the internet). Such an infection might be not direct – the malware (like trojans or viruses) might first infect less-protected systems and advance to the crucial systems (Axelrod, 2017). Cars might also be attacked by putting an infected CD into the CD player, which could automatically download malware (Checkoway et al., 2011). Resulting attack might manifest as a crash of the system which drives the car. Car sharing companies often make people use smartphones to access the car; attacking the smartphone or the communication between the phone and car might be a way the attacker might get into the car (Haas and Möller, 2017). Malware installed through social engineering or car-sharing might lead also to attacks happening during the time when no one is present in the car, so the car might be stolen.

Cybersecurity experts offer plenty of solutions to ensure better AV cybersecurity. Countries should strengthen control of companies producing AV (Lim and Taeihagh, 2018), standardize AV technologies (He et al., 2017), and introduce cybersecurity ranking measures (Burzio et al., 2018). Companies should control products from their suppliers (Parkinson et al., 2017). Different types of communication (Messnarz et al., 2017) and layers of intrusion detection system (Straub et al., 2017) should be mutually independent and each component should have its own firewall (Rizvi et al., 2017). Security system should be often actualized and CAN protected from scanning (Lim et al., 2017). There might be installed chips controlling behavior and temperature of different hardware components to be able to signalize cyberattack (Lima et al., 2016). Also, user interface could be changed to make people more often agree with the cybersecurity-enhancing options (Stavova et al., 2018). If possible, all these mitigation measures should be used simultaneously (Al Mamun et al., 2018).

## Human Factor in Autonomous Vehicle Cybersecurity

Psychologists might help to improve cybersecurity in various ways. People differ in their ability to correctly assess the cybersecurity risk. As found by Yan et al. (2018), 23% of people correctly handle less than half of cybersecurity scenarios; only 4% can handle more than 90% of scenarios. Cybersecurity awareness is a critical issue for AV drivers; therefore, it will be necessary to increase knowledge about cybersecurity for these drivers. Several researchers investigated the characteristics of people with inadequate cybersecurity skills. On the internet, people are prone to behaving in a more risky fashion toward cybersecurity if they are more extraverted, addicted to the internet, impulsive, and less conscientious (Hadlington, 2017). Those who more often use a workplace computer for non-work purposes have less internet security awareness (Hadlington and Parsons, 2017). Men have more experience with cybersecurity than women (Anwar et al., 2017). Anxious people are less successful in detecting a cyberattack (Welk et al., 2015). The characteristics of people with riskier behavior toward AV cybersecurity are yet unknown. The goal for human-factor researchers is to identify the people who are the most vulnerable in AV cybersecurity scenarios, to identify the kinds of scenarios they fail in, and to develop targeted educational materials.

Risky cybersecurity behavior is connected to the over-trust of automated technologies (Noy et al., 2018). When the driver trusts their car too much, it is more prone to attack (Parkinson et al., 2017). An open research question is how to explain these cybersecurity issues to the public and which factors influence the correct recall of this information. People do not understand cybersecurity issues better when the problem is explained metaphorically. A disease-risk metaphor and criminal behavior metaphors do not increase understanding, and a physical assault metaphor worsens it (Brase et al., 2017). The ability to memorize cybersecurity news is moderated by the cyber anxiety of the person: people with higher anxiety related to cyberattacks are bad at retaining cybersecurity-related news (Cheung-Bluden and Ju, 2016).

Engaging in behaviors to enhance cybersecurity is related to the belief that these behaviors are effective and that the cost of the engagement (e.g., time loss) will be minimal (Blythe and Coventry, 2018). People who follow cybersecurity instructions are those who consider ignoring them to be more risky (Fagan and Khan, 2018). Informing about the risks connected to cyberattacks could be the way to make people to behave more securely.

The level of multitasking in which a driver engages might influence the effectiveness with which they are able react to cybersecurity breaches. People who multitask are prone to risky cybersecurity behavior (Hadlington and Murphy, 2018). Distraction leads to less success in identifying malicious attacks (Kortschot et al., 2018). The ability to react appropriately to a cybersecurity breach is problematic, especially in a transition period, when drivers are not yet used to AV and cognitive overload will be common. Based on research in this field, when AV drivers have to react to unexpected events, they have a wide range of reaction times and the ability to react differs (Gold et al., 2013; Dixit et al., 2016; Dogan et al., 2017). And cybersecurity issues will be more abstract and difficult than real-environment problems. Furthermore, because it is expected that driving skills will decrease with the use of the AVs, number of cybersecurity attacks might rise with time. This raises a question about what amount of distraction and multitasking is acceptable for an AV driver to be able to react to driving issues, not only in general, but especially in terms of cyberattacks. Researchers should provide an answer so that authorities can set appropriate policy.

The frequency of cyberattacks influences human ability to defend against them. Attacks based on social engineering like fishing might be successful only when they are rare. In email communication, when attacks are rare, people are more likely to mistakenly open malicious email. When attacks are more frequent, people trust email less and make fewer mistakes (Sawyer and Hancock, 2018). Researchers should look for a similar relationship for AV usage. They should find what time delay between cyberattack attempts is enough for a driver to lose the ability to react appropriately to cyberattacks, and offer solutions for how to improve drivers’ reaction ability. Related issue is the human tendency to lose attention during monotonous task like driving without cognitive involvement of a driver (Saxby et al., 2013) and mitigation of its consequences for readiness to react during cyberattack.

Autonomous vehicles need the authentication of the user (e.g., a password or a passphrase; Juang and Greenstein, 2018). Authentication should be safe; however, it should also be quick and easy to understand so that user can proceed with the proper action quickly. Authentication should also be inclusive and possible for blind people and people with other kinds of impairment. This is a difficult goal (Still et al., 2017) and requires the involvement of human-factor researchers.

Another open issue is how people behave during an AV cyberattack. A cyberattack induces stress in person whose device is attacked (Canetti et al., 2017). When people know that the attacker has adapted to their behavior, they start to behave more randomly (Moisan and Gonzalez, 2017). It is important to research the ways that the car can effectively communicate both the information about the active cyberattack and the appropriate response to an inexperienced driver (Parkinson et al., 2017).

Working in cybersecurity is a very demanding job. The selection of appropriate employees improves cybersecurity culture in an organization and leads to better security decisions (Parsons et al., 2015). Employees should be good team workers and system thinkers. They should also have the necessary technical skills, be able to communicate information to common people, be determined to fulfill their duty, be able to learn continuously (Marble et al., 2015; Dawson and Thomson, 2018), and be well informed about their company’s cybersecurity policy (Li et al., 2019). Employees with higher threat awareness and countermeasure awareness perform better in cybersecurity tasks (Torten et al., 2018). Experience from nuclear power plant personnel selection shows that people hired for different positions need different set of skills (Schumacher et al., 2011). Teams that contain people with hostile personality traits perform better in solving cybersecurity scenarios (Cowley et al., 2015), while people with interest in a cybersecurity career tend to have higher self-efficacy and a rational decision-making style (Bashir et al., 2017). Buchler et al. (2018) show that the best performing cybersecurity teams have members who are specialized in specific cybersecurity roles. Specialization brings higher requirements for employee selection. Human-factor researchers should develop procedures that will lead to the effective selection of employees for companies dealing with AV communication infrastructure to ensure maximum safety. Additionally, the percentage of women currently working in cybersecurity in different regions does not exceed 14%, and in Europe it is only 7%, which might be due either to the discrimination women feel in cybersecurity workplaces or bias in selection procedures (Poster, 2018). The selection of employees should address this problem and become less gender biased.

Companies that will be controlling AV cybersecurity must be sure they can trust all of the people in their organizations (Henshel et al., 2015). They should carefully monitor their employees for abuse of their positions (Evans et al., 2016). Hadlington (2018) provides additional suggestions to guard against malicious insiders in an organization – using only trusted connections (e.g., Wi-Fi), using strong passwords, regularly updating software, and limiting personal information shared online. Greitzer and Frincke (2010) suggest keeping tabs on employee stress, disgruntlement, disengagement, disregard for authority, confrontational behavior, dependability, absenteeism, and performance (nevertheless, confrontational behavior might be beneficial for those who should solve and discover cyberattacks – see Cowley et al., 2015). Knowing their personality characteristics and these metrics, the online behavior of employees should be watched and the risk of a cybersecurity breach assessed. When risky behavior is anticipated, the employee should be released from their duties. Greitzer and Frincke (2010) consider such an assessment and its impact on an employee’s career to be ethically questionable. Nevertheless, the development of such procedures might become necessary when cyberattacks on the AV infrastructure result in deaths. Such monitoring might be demanding for Human Resources departments and it needs the HR personnel to be continuously educated in these issues (Dreibelbis et al., 2018).

Knowing the motivations and characteristics of attackers might also help to prevent future attacks. King et al. (2018) think that attackers might be characterized by low social status, hyperactivity, socialization toward rule-breaking behavior, and the dark triad personality traits (psychopathy, narcissism, and machiavellianism). These characteristics might help to develop methods for attacker identification (e.g., analyzing their online social network profiles, methods for successful deterrence of attackers; Lindsay, 2015). Developing specific methods for AV attackers is a goal for future researchers. Such methods would differ for different types of attackers. According to Derrick et al. (2016) attackers could be thieves, organized criminals, political activists, terrorists, foreign government, or vehicle owners themselves. For example, groups of hacktivists might be motivated to participate in cyberattacks to develop a strong identity and the ethos for their group (Thackray et al., 2016). Developing methods to damage the identity of groups that attack AV might help to maintain security.

Cultural differences and specifics should be considered when discussing AV cybersecurity issues. People from cultures with higher uncertainty avoidance, where self-control is preferred over personal desire, might be more prone to ideological indoctrination, such that attack consequences might be exacerbated by the ideological motivation of the attacker. Attackers from cultures with higher long-term orientation might plan more sophisticated attacks (Henshel et al., 2016). These factors should be considered when designing cars for countries with such cultures.

## Concluding Remarks

Cybersecurity research that concerns human factors is still an emerging field (Bordoff et al., 2017). The basic concepts, like cybersecurity culture, are not yet clearly defined (Gcaza and von Solms, 2017). Given that fully autonomous traffic does not yet exist and non-connected autonomous cars exist only in some parts of the world, research on how people behave toward autonomous driving is nearly absent. Psychological researchers might provide a large improvement in the security of AV by investigating these phenomena (Wiederhold, 2014).

There are five types of issues to be researched. First, the characteristics of people vulnerable to AV cybersecurity error and the types of scenarios they fail in. Second, the ways to effectively educate people to improve their AV cybersecurity skills. Third, how to effectively select and work with employees of companies in charge of AV cybersecurity. Fourth, how to lower the motivation of attackers (see Table 1). And, fifth, how the decrease of driving skills as a consequence of autonomous driving will affect the driver’s ability to react to cybersecurity issues (nevertheless, it seems that decrease of driving skills is not that large even if person does not drive for long time – see Trösterer et al., 2016).

**Table 1:** How might human factor researchers improve the cybersecurity of autonomous vehicles (AV).

Security vulnerability	Research goal	Benefit
Characteristics of people who are vulnerable to AV cybersecurity failure is unknown	Identify groups of people who are likely to perform badly in an AV cybersecurity scenario	Vulnerable groups may be targeted by a promotional campaign
Factors that influence human AV cybersecurity performance are not completely known	Identify factors that enhance AV cybersecurity performance	Possible to set policy to increase these factors
Over-trust of AV	Identify groups of people likely to over-trust AV security	Vulnerable groups may be targeted by an educational campaign
AV cybersecurity is problematic and not correctly understood by laypeople	Identify effective ways to explain AV cybersecurity	Educational campaign will increase knowledge
Acceptable multitasking is unknown	Identify acceptable level of multitasking to be able to react to an AV cybersecurity breach	Possible to set policies regarding multitasking for AV
Time when AV cybersecurity defense capability decreases is unknown	Identify period needed to review information about AV cybersecurity	Possible to remind driver after this period
How people behave during specific AV cyberattacks	Understand weak points of people’s reactions to cyberattacks	Develop techniques to help laymen during an attack
People working in AV cybersecurity should be able to work in a demanding job	Understand requirements of AV cybersecurity jobs	Develop strategies to correctly select employees for AV cybersecurity
Employees in AV cybersecurity might become attackers or help attackers	Identify detectable behavior changes typical for renegades	Possible to remove risky employees
Characteristics of AV attackers are unknown	Identify who attacks AV and why	Set policies to decrease the motivation of attackers

It is impossible to reach complete cyber safety for AV. Cyberattacks will always happen and some of them will be successful. Therefore, an effective strategy is to not try to eliminate all cyberattacks, but to accept their existence and prepare to react to their consequences (Lin et al., 2016). Some countries, like the United States, China, and Singapore, have already established laws for cybersecurity issues (Taeiagh and Lim, 2018); other countries should follow and prepare for future.

Human-factor researchers should note that changes made to enhance AV cybersecurity might not always increase traffic safety. Macher et al. (2017) gives an example of a situation where a steering wheel is blocked in a dangerous situation. This is safe from a cybersecurity point of view, because the attacker cannot change the steering wheel position. Nevertheless, it is not safe from the position of traffic safety – when a steering wheel is blocked, the driver cannot react appropriately. Researchers should consider these types of situations when designing AV security and think about traffic safety more globally. It should be also noted that cybersecurity threat might be overestimated: Quigley et al. (2015) analyzed texts written by cybersecurity experts and found that many of them use rhetorical techniques to make this threat look larger.

Finally, most of the research suggested here cannot yet be fully conducted, different research issues will be important in different stages of AV automation (see Table 2). Research on human behavior when dealing with AV needs to investigate the experience of autonomous driving on common people. Fully AV are not yet widespread, so finding research participants is difficult. Research concerning AV cybersecurity will be more suitable in the future when people will use AV regularly. Nevertheless, researchers should try their best to find what is possible now, because governments and companies should be prepared for the future.

**Table 2:** Priority of different human-factor-research related issues in AV cybersecurity in various levels of AV automation as defined by SAE International (2014).

SAE level of automation	(0) No automation	(1) Driver assistance	(2) Partial automation	(3) Conditional automation	(4) High automation	(5) Full automation
**Research issue:**						
Cybersecurity failure prone people’s characteristics	Small	Small	Middle	High	High	Middle
Ways to increase cybersecurity performance	Small	Small	Small	High	High	High
Overtrust to AV	Small	Middle	Middle	High	High	High
Laypeople education	Middle	Middle	Middle	Middle	Middle	Middle
Multitasking acceptability	Small	Middle	Middle	High	High	Small
Cybersecurity defense capability decrease	Small	Small	Middle	High	High	High
Behavior during cyberattacks	Small	Small	High	High	High	High
AV infrastructure companies job requirements	Small	Small	Small	High	High	High
Characteristics of AV cyberattackers	Small	Small	Middle	High	High	High

## Author Contributions

All authors listed have made a substantial, direct and intellectual contribution to the work, and approved it for publication.

## Conflict of Interest Statement

The authors declare that the research was conducted in the absence of any commercial or financial relationships that could be construed as a potential conflict of interest.
